# Lysosomal Exocytosis, Exosome Release and Secretory Autophagy: The Autophagic- and Endo-Lysosomal Systems Go Extracellular

**DOI:** 10.3390/ijms21072576

**Published:** 2020-04-08

**Authors:** Sandra Buratta, Brunella Tancini, Krizia Sagini, Federica Delo, Elisabetta Chiaradia, Lorena Urbanelli, Carla Emiliani

**Affiliations:** 1 Department of Chemistry, Biology and Biotechnology, University of Perugia, Via del Giochetto, 06123 Perugia, Italy; sandra.buratta@unipg.it (S.B.); brunella.tancini@unipg.it (B.T.); krizia.sagini@ous-research.no (K.S.); federica.delo@studenti.unipg.it (F.D.); 2 Department of Molecular Cell Biology, Institute for Cancer Research, Oslo University Hospital-The Norwegian Radium Hospital, 0379 Montebello, Oslo, Norway; 3 Department of Veterinary Medicine, University of Perugia, Via S. Costanzo 4, 06126 Perugia, Italy; elisabetta.chiaradia@unipg.it; 4 Centro di Eccellenza sui Materiali Innovativi Nanostrutturati (CEMIN), University of Perugia, Via del Giochetto, 06123 Perugia, Italy

**Keywords:** lysosomal exocytosis, exosomes, extracellular vesicles, secretory autophagy, autophagosomes, amphisomes, unconventional protein secretion

## Abstract

Beyond the consolidated role in degrading and recycling cellular waste, the autophagic- and endo-lysosomal systems play a crucial role in extracellular release pathways. Lysosomal exocytosis is a process leading to the secretion of lysosomal content upon lysosome fusion with plasma membrane and is an important mechanism of cellular clearance, necessary to maintain cell fitness. Exosomes are a class of extracellular vesicles originating from the inward budding of the membrane of late endosomes, which may not fuse with lysosomes but be released extracellularly upon exocytosis. In addition to garbage disposal tools, they are now considered a cell-to-cell communication mechanism. Autophagy is a cellular process leading to sequestration of cytosolic cargoes for their degradation within lysosomes. However, the autophagic machinery is also involved in unconventional protein secretion and autophagy-dependent secretion, which are fundamental mechanisms for toxic protein disposal, immune signalling and pathogen surveillance. These cellular processes underline the crosstalk between the autophagic and the endosomal system and indicate an intersection between degradative and secretory functions. Further, they suggest that the molecular mechanisms underlying fusion, either with lysosomes or plasma membrane, are key determinants to maintain cell homeostasis upon stressing stimuli. When they fail, the accumulation of undigested substrates leads to pathological consequences, as indicated by the involvement of autophagic and lysosomal alteration in human diseases, namely lysosomal storage disorders, age-related neurodegenerative diseases and cancer. In this paper, we reviewed the current knowledge on the functional role of extracellular release pathways involving lysosomes and the autophagic- and endo-lysosomal systems, evaluating their implication in health and disease.

## 1. Introduction

**Figure 1:**
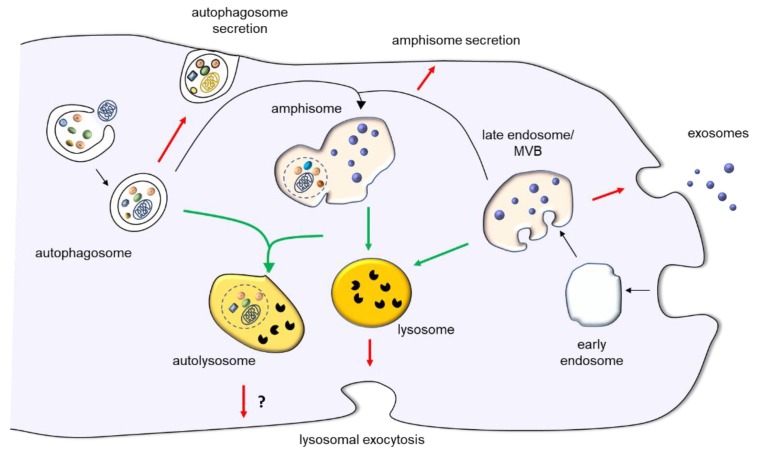
Overview of lysosomal exocytosis, exosome release and autophagy-dependent secretory pathways. Lysosomal exocytosis leads to the secretion of lysosomal content upon lysosome fusion with plasma membrane. Exosomes originate from the inward budding of late endosome membrane, which originates MVBs. They are either released extracellularly upon exocytosis or degraded into lysosomes. Autophagy is a cellular process leading to sequestration of cytosolic cargoes for their degradation within lysosomes. However, the autophagic machinery is also involved in autophagy-dependent secretion of autophagosomes. In addition to merging with lysosomes or plasma membrane, autophagosomes can also fuse with late endosomes/MVBs to produce amphisomes. In turn, amphisomes can either fuse with lysosomes to degrade their content or with plasma membrane. The red arrows indicate fusion with plasma membrane, the green arrows, fusion with lysosome, and the black arrows, pathways leading to organelle maturation and to the intersection between autophagic and endocytic pathway.

Lysosomes are terminal degradative organelles whose functions are fundamental to maintain cell homeostasis, but there is evidence that the content of lysosomes and of organelles of the autophagic- and endo-lysosomal system can be poured out the cell, contributing to cellular clearance and cell-to-cell communication. Lysosomal exocytosis leads to the secretion of lysosomal content upon fusion of lysosomes with the plasma membrane. This allows to accomplish important functions, such as plasma membrane repair and tissue remodeling. Current investigations also provide evidence that organelles delivering cell material to lysosomes for degradation, such as autophagosome and endosomes, can change their destination from fusion with lysosomes to fusion with plasma membrane for extracellular release. From a functional point of view, this alternative route appears to be not only an additional manner to dispose waste, but a process finely tuned which is relevant for the unconventional protein secretion of signaling molecules, for the release of vesicles originating in late endosomes (exosomes), which are now considered an additional manner of cell-to-cell communication, and for immune surveillance of pathogens (Figure 1). In this review, we summarize the current knowledge on lysosomal exocytosis, exosome release and secretory autophagy to shed light on the functional role and pathological implications of the extracellular release of lysosomes and other organelles of the autophagic- and endo-lysosomal system.

## 2. Lysosomal Exocytosis

In recent decades, it has been clearly demonstrated that lysosomes can accomplish a secretory pathway known as lysosomal exocytosis [1,2]. At first, lysosomal exocytosis was regarded as a function of specialized secretory cells, namely hematopoietic cells, containing a peculiar type of lysosomes, which acquired the competence of regulated secretory organelles (secretory lysosomes). However, many successive studies have shown that all cell types can secrete their lysosomal content in response to different stimuli, upon fusion of lysosomes with the plasma membrane [1,3,4]. It is now undoubtedly ascertained that different specialized cell types carry out lysosomal exocytosis to accomplish their biological function following specific stimuli. Lysosomal exocytosis is a ubiquitous Ca^2+^-regulated mechanism which plays a crucial role in several physiological processes, such as plasma membrane repair [5,6], bone resorption by osteoclasts [7], melanocyte function during pigmentation [8], immune response against parasitic attack [9,10] and antigen presentation [3]. More recently, evidence has been produced that lysosomal exocytosis is involved in ATP release in response to different stimuli in the CNS. For instance, lysosomal exocytosis may contribute to intercellular signaling in astrocytes by promoting the extracellular release of ATP, which is required for Ca^2+^ wave propagation [11]. Ca^2+^-dependent lysosomal exocytosis appears to be responsible for the intracellular ATP decrease, which is observed in astrocytes under oxidative stress induced by H_2_O_2_. As a high concentration of extracellular ATP is toxic to neurons [12], lysosomal ATP release may exacerbate at tissue level the cellular damage induced by oxidative stress [13]. In microglia, ATP released by lysosomal exocytosis has been reported to contribute to the directional migration of remote microglia. Authors suggested the existence of a positive feedback mechanism mediated by ATP-induced lysosomal ATP release, which establishes a long-range extracellular ATP gradient promoting remote microglia chemotaxis and inducing the recruitment of distant microglia at the site of injury [14].

As mentioned above, lysosomal exocytosis also plays a crucial role in plasma membrane repair in all cell types. Restoration of plasma membrane integrity after injury is of fundamental importance to ensure plasma membrane selective permeability and hence cell homeostasis maintenance. Upon plasma membrane injury, lysosomes located near the wounded site quickly migrate and fuse with the plasma membrane, efficiently resealing the damage [5]. It has been shown that the rapid local increase of Ca^2+^ caused by its influx through the plasma membrane at the wound site induces a conformational change in synaptotagmin VII, a ubiquitous lysosomal membrane calcium-sensor containing two Ca^2+^-binding C2 domains. This leads to synaptotagmin VII interaction with preformed SNARE (soluble N-ethylmaleimide sensitive factor receptor) complexes and plasma membrane phospholipids, which in turn drives the formation of a fusion pore and the release of lysosomal content into the extracellular matrix [6,15]. However, this process is important not only for plasma membrane repair but also for specific membrane remodeling mechanisms. For instance, during phagocytosis, macrophages form structures called pseudopods to surround and engulf large cellular particles, such as apoptotic bodies. This requires a large amount of intracellular membranes which is mostly supplied by exocytosis of endosomes and lysosomes [16]. Lysosomal exocytosis also plays a role in neurite outgrowth, which requires exocytosis of late endosomes/lysosomes for the elongation of the developing neuronal processes [17]. Recently, Naegeli et al. reported that localized lysosomal exocytosis directed by netrin receptor UNC-40 serves to expand basement membrane openings during C. elegans anchor cell invasion. The authors showed that lysosomal exocytosis can provide a local lysosome membrane source necessary to build up the large protrusion that promotes tissue invasion [18]. As it has been previously reported that cancer cells form single large protrusions to breach basement membrane and cross tissue barriers [19,20], this work opened an intriguing question about the possibility that localized lysosomal exocytosis may be a key step during cancer metastasis.

From a molecular point of view, the mechanistic organization of lysosomal exocytosis implies the recruitment of lysosomes to the proximity of the cell surface and the subsequent Ca^2+^-dependent fusion of pre-docked lysosomes with the plasma membrane, leading to the release of the lysosomal content into the extracellular milieu [1,21]. The main steps of the process have been elucidated. Upon stimulation, lysosomes translocate from the perinuclear region of the cell to the plasma membrane by associating with the microtubule-dependent motor protein kinesin [5,22]. The fusion process starts with the formation of a trans-SNARE complex among the vesicle-associated membrane protein 7 (VAMP7), a v-SNARE protein located on the lysosomal surface, and syntaxin-4 and the synaptosome-associated protein of 23 kDa (SNAP23), which are present on the plasma membrane [23]. The trans-SNARE complex formation allows the two membranes to come closer together and lysosomes to dock to the plasma membrane. Finally, local intracellular Ca^2+^ increase triggers the direct fusion of lysosomes with the plasma membrane by promoting the interaction between synaptotagmin VII and the pre-formed trans-SNARE complex [5,24]. Several small GTPases Rab proteins also take part in the process and it has been recently reported that two of them, Rab3a and Rab10, are essential for lysosomal exocytosis and plasma membrane repair [25].

Lysosomal exocytosis is a fine regulated process. Transient receptor potential mucolipin 1 or mucolipin 1 (TRPML1 or MCOLN1), the principal Ca^2+^ release channel in the lysosome, is a key mediator of lysosomal exocytosis and is under the control of the transcription factor EB (TFEB), a master gene regulating lysosome biogenesis and autophagy [2]. TFEB controls lysosomal exocytosis inducing local Ca^2+^ increase trough the transcriptional activation of the lysosomal cation-channel MCOLN1 and triggering the final fusion of lysosomes with the plasma membrane. Moreover, TFEB regulates lysosomal exocytosis by transcriptionally activating tethering factors and proteins involved in lysosomal dynamics, docking and fusion with the plasma membrane [26]. Therefore, TFEB regulates lysosomal exocytosis by promoting first the lysosome recruitment and docking to the plasma membrane, then the Ca^2+^-mediated fusion event through the activation of the TRPML1 channel.

The proper activity of lysosomal exocytosis has important pathological implications. For instance, activation of lysosomal exocytosis has been proven to promote cellular clearance in cell and animal models of various lysosomal storage diseases (LSDs). LSDs are a group of inherited metabolic disorders characterized by the intralysosomal accumulation of undigested materials. The lysosomal storage results in lysosomal dysfunction and cell death, culminating in multisystemic pathological symptoms often associated with neurodegeneration [27]. Lysosomal exocytosis by TFEB overexpression was able to promote cellular clearance in glia-differentiated neuronal stem cells isolated from mouse models of multiple sulfatase deficiency and mucopolysaccharidoses type IIIA, two LSDs characterized by lysosomal glycosaminoglycan accumulation [2]. Similar results were also obtained by using cells from a mouse model of neuronal ceroid lipofuscinoses (Batten disease) and fibroblasts from a patient affected by glycogenosis type II (Pompe’s disease) [2]. Moreover, clearance of stored materials was also obtained in in vivo studies upon viral-mediated TFEB gene transfer in mouse models of multiple sulphatase deficiency [2] and Pompe’s disease [28]. Therefore, TFEB overexpression induces lysosomal exocytosis and promotes cellular clearance in different types of LSDs independently of the nature of stored materials. However, TFEB overexpression failed to induce cellular clearance in a mucolipidosis IV model characterized by mutations in the MCOLN1 gene, suggesting that TFEB-mediated lysosomal exocytosis and clearance may occur through the activation of the Ca^2+^ channel TRPML1 [2]. Therefore, an alternative way to promote lysosomal exocytosis may be trough the activation of TRPML1. In fact, specific TRPML agonists such as SF-51 and ML-SA1 have been proven effective to promote cellular clearance in in vitro studies [29,30,31]. Based on these findings, induction of lysosomal exocytosis to promote cellular clearance has been proposed as an alternative therapeutic strategy to treat not only LSDs, but also other pathological conditions characterized by undigested substrates accumulation and overloaded dysfunctional lysosomes, such as several common neurodegenerative disorders [2,32]. For instance, promising results have been recently obtained by increasing lysosomal exocytosis with lysosomal Ca^2+^ channel TRPML1 agonists in iPSC-derived dopaminergic neurons from PARK9 mutated Parkinson’s disease patients [33]. Authors showed that lysosomal exocytosis is involved in clearing intracellular α-synuclein in human dopaminergic neurons and that mutations in PARK9 contribute to the α-synuclein storage. Drug-induced increase of lysosomal Ca^2+^ level rescued defective α-synuclein secretion and alleviate its accumulation in patient neurons, suggesting that lysosomal exocytosis may represent a potential therapeutic target in Parkinson’s disease and other related synucleinopathies [34]. Nevertheless, although lysosomal exocytosis enhancement can represent a very appealing therapeutic strategy also due to its wide potential applications, the effect on neighboring cells of a large amount of toxic materials released in the extracellular space must be carefully assessed [34].

In summary, the quantity and specificity of the physiological processes involving exocytosis of lysosomes in different cell types highlights another peculiar function of lysosomes which deserves particular attention for its possible implications both in physiological and pathological conditions. Notably, lysosomal exocytosis is a Ca^2+^-dependent process under the control of the master gene TFEB and thus it may represent a coordination point between catabolic and secretory processes in response to cellular needs [2]. Finally, lysosomal exocytosis can also be regarded as a potential therapeutic target for those pathologies characterized by lysosomal storage and dysfunction, namely LSDs and age-related neurodegenerative diseases.

## 3. Exosomes

Exosomes are a subset of extracellular vesicles (EVs) originating from the endosomal system and released in the extracellular milieu. Whereas EVs is a term generally used to describe the entire pool of membrane-limited particles released by cells, the term exosome indicates vesicles with a small size (<150 nm), enriched in endosomal proteins. The dimension and the cargo of exosomes are reminiscent of their origin, that begins with the invagination of late endosomal membrane and ends with the production of intraluminal vesicles (ILVs) within the organelle. These peculiar endosomes, called multivesicular bodies (MVBs), can fuse with plasma membrane to release ILVs into the extracellular space, originating exosomes [35,36]. MVBs can also fuse with lysosomes, but the mechanisms responsible for MVBs to reach the membrane or the lysosome are still unclear, although it has been reported that MVBs containing high level of cholesterol are more prone to fuse with plasma membrane releasing exosomes [37]. Notably, during their biogenesis exosomes selectively capture cell-specific macromolecules (proteins, lipids, RNAs or even DNA), which became part of the exosomal cargo and are responsible of their functions [38].

A methodological limitation in studying exosomes and determining their content or biological role is that there are no available methods which allow to separate exosomes from other types of small EVs [36]. For this reason, the term exosome has been improperly used in an enormous number of studies to describe EVs of small size isolated using differential centrifugation protocols including a final high-speed ultracentrifugation step [39,40]. This protocol, as well as other protocols based on size-exclusion chromatography, allows the isolation of vesicles with a precise size/density, but is not able to efficiently separate exosomes from vesicles of non-endosomal origin (e.g., microvesicles derived from the outward budding of plasma membranes) or non-vesicular structures, such as newly identified exomeres [41,42]. Indeed, Kowal and coworkers [40] demonstrated the presence of exosomal and non-exosomal subpopulations within small EVs isolated by ultracentrifugation followed by flotation into density gradients. Therefore, it has to be considered that the majority of the studies reports the properties of small EVs enriched in exosomes and not of purified exosomes.

The first studies, dating back to the 1980s, considered the release of exosomes an alternative route used by cells to eliminate unnecessary material [43]. This role has been also highlighted by recent data demonstrating that regulation of exosome secretion is important to maintain cellular homeostasis [34,44,45]. However, it has currently become clear that exosomes play important roles in intercellular communication and are implicated in several pathophysiological processes, including inflammation, tumor invasion, immune response, differentiation [46]. This evidence derives from many studies that showed that these functions are mediated by the cargo of exosomes (nucleic acids, proteins and lipids), that is transferred in an active form from releasing to recipient cells [46].

Exosome biogenesis includes several steps and many of the components involved are also implicated in other pathways of the vesicular trafficking. ILVs formation can be considered the first step of the process and can be initiated either by ESCRT (Endosomal sorting complex required for transport)-dependent or ESCRT-independent mechanisms. The involvement of ESCRT machinery and their accessory proteins in ILV formation is confirmed by evidences showing that the silencing of selected individual ESCRT components affects exosome size, quantity and protein cargo [47]. ESCRT machinery is composed of four protein complexes (ESCRT 0, I, II, III), that along with accessory proteins (ALIX, Tsg101, VSP4) associate onto endosomal membrane in a coordinated manner, regulating cargo selection and ILV formation [41]. Firstly, ESCRT 0 is recruited to the endosomal membrane by monoubiquitinated transmembrane proteins, then ESCRT I and II promote the invagination of the membrane domain containing these complexes and finally ESCRT III completes the scission of the membrane, generating ILVs [46]. The formation of ILVs and the selection of the exosome molecular cargo can be also regulated by an ESCRT-independent mechanism involving membrane microdomain enriched in tetraspanins [48]. These are a family of membrane proteins that are considered the best currently available markers for exosomes [42]. The role of these proteins in exosome biogenesis is supported by studies demonstrating that changes in the expression level of CD9, CD81 and CD63 affect the total amount of secreted exosomes [49,50,51]. The expression of tetraspanins, i.e., Tspan8 and CD63, also affects the molecular composition of exosomes [36]. Profound changes in membrane composition of MVBs occurring during ILV formation and MVB fusion with the plasma membrane underline the important role played by lipids in exosome biogenesis. Indeed, several studies demonstrate that specific lipids and lipid-related enzymes participate to this process [52]. Specific lipid classes involved in ILV formation are ceramide, lysophospholipids and phosphatidic acid (PA), whose accumulation in membranes promote the formation of lipid microdomain and membrane invagination. In several cell types, it has been demonstrated that the inhibition of ceramide production reduces exosome secretion and a role in this event is played by sphingomyelinase 2, an enzyme generating ceramide from sphingomyelin [53,54,55]. PA is a phospholipid characterized by a small and negative polar head. PA increased level during membrane rearrangement generates negative membrane curvature [56]. Coherently, the PA-producing enzyme such as phospholipase D [57,58] and diacylglycerol kinase [59,60], regulate the release of exosomes in several cell lines. Altogether, these studies suggest that MVB subpopulations using different machinery for exosome biogenesis exist in different cell types and/or co-exist in the same cell type [46].

Several physiological and pathological stimuli could induce the release of exosomes. Among them, lysosomes play a role in the regulation of exosome biogenesis and release. As previously reported, the molecular mechanisms determining the fate of the MVBs versus plasma membrane or lysosomes are not defined. In any case, a link has been demonstrated between the two alternative pathways. The treatment of cells with drugs, i.e., bafilomycin A or chloroquine, that alkalinize lysosomal pH impairs lysosomal function and increases the level of released EVs [61,62]. This evidence suggests that EV release could be a compensatory event for lysosomal impairment or overload with unnecessary/damaged molecules. An increase of EV release was observed in Niemann Pick Type C (NPC), an LSD characterized by lysosomal accumulation of cholesterol and sphingolipids due to mutation in the NPC1 gene [63,64]. These studies support the hypothesis that lysosomal dysfunction induced by stored materials drives MVB release into the extracellular space to maintain cellular homeostasis [34]. Moreover, in NPC the molecular cargo of exosomes was also modified in a gene mutation-dependent manner [63,64]. Even if a possible role of EVs as an alternative disposal mechanism has been studied in NPC disease, it is unclear whether takes place also in other LSDs. Instead, this phenomenon is evident in neurodegenerative disorders (e.g., Alzheimer’s disease, Parkinson’s disease, Huntington’s disease) characterized by an impairment of the autophagy-lysosomal pathway. The inhibition of lysosomal-related functions renders the cell unable to degrade the overloaded defective proteins. Several studies demonstrated that many of these proteins are released via EVs to compensate their reduced degradation inside the cell [65]. In this context, an impairment of exosome biogenesis and secretion in neurodegenerative diseases caused by genetic mutations has been demonstrated [66]. These vesicles also participate to the propagation of neurodegeneration by spreading toxic molecules, such as misfolded proteins, miRNA or inflammatory mediators [67]. An important consequence of this event is the potential role of exosomes as biomarkers. The presence of disease-related molecules, namely miRNAs and proteins, in exosomes isolated from blood and cerebrospinal fluid of patients, provides the rationale for their use as diagnostic tool for neurodegenerative diseases [68]. Furthermore, exosomes might be used as drug-delivery vehicles for treatment of neurological disorders, as they can cross the blood brain barrier and deliver pharmacologically active molecules, protecting them from degradation [69]. As for example, exosomes carrying specific siRNAs or exosomes derived from mesenchymal stem cells have been shown to be beneficial in Parkinson’s disease and in other pathological conditions [70,71].

Interestingly, the release of exosomes could depend on lysosomal exocytosis. As previously described, late endosome/MVBs can fuse with lysosomes or with plasma membranes. Although fusion with lysosomes leads to degradation of endosomal content, recent evidence demonstrates that exosomes are present into lysosomes, where they are protected from degradation and are released by lysosomal exocytosis [72,73]. In cancer cells, the downregulation of the lysosomal sialidase NEU1 induces the accumulation at the lysosomal membranes of over-syalilated LAMP1, that in turn promotes lysosomal exocytosis of hydrolases and exosomes [72]. Authors proposed that in tumor cells the enhanced lysosomal exocytosis of hydrolytic enzymes together with exosomes promotes invasion and degradation of extracellular matrix and may condition neighboring cells to become migratory cells via signals mediated by released exosomes [72]. Thus, the inhibition of lysosomal exocytosis could be a therapeutic strategy useful to reverse invasiveness and chemoresistance in cancer cells. The possibility that a part of exosomes derives from lysosomes and is released by lysosomal exocytosis was confirmed by a recent study demonstrating that the secretion of exosomes from adipocyte is mediated by TRPML1-dependent lysosomal exocytosis [73].

The uptake of EVs by acceptor cells has been reported to occur through different mechanisms. EVs can fuse with plasma membrane of the recipient cell releasing cargo directly into the cytosol or can be internalized by endocytosis [41]. Once internalized, EVs may be recycled and re-secreted or can interact with endo-lysosomes delivering their cargo for degradation. In PC12 cells, Thian and coworkers demonstrated that EVs are actively transported along cytoskeleton toward the perinuclear region, where they mostly fused with lysosomes [74]. Several studies demonstrate that EVs exert their biological effect after their internalization into lysosomes [64]. An example is represented by the demonstration that exosomes derived from SW480 cancer cells enter recipient cells by endocytosis, localize into lysosomes and induce cell migration activating the MAP kinase pathway [75]. Altogether, these results highlight the central role of lysosomes in the cellular and molecular mechanisms regulating exosome release, uptake and function.

## 4. Secretory Autophagy

Lysosomes contain hydrolases that participate in the digestion of substrates that come either from outside or from inside the cell. Autophagy has been initially defined by Christian De Duve as a self-eating process, i.e., a process allowing cell to digest and recycle unnecessary or harmful intracellular components, in opposition with heterophagy, a process allowing cells to degrade extracellular material [76]. Although once considered an exclusive degradative process, it is now acknowledged that autophagy has also important signaling and metabolic function, being able to finely tune the degradation of specific substrates according to cellular needs.

### 4.1. Autophagy: An Overview

The autophagic process can be distinguished by other cellular degradative processes, such as proteasomal digestion, because it is able to digest not only cytosolic proteins, but also damaged organelles, namely mitochondria and peroxisomes [77,78,79]. The autophagy machinery delivers these components into lysosomes, where hydrolases break down macromolecules into their constituents, recycling them for cell needs. The engulfment of cytosolic components is a distinctive feature of autophagy, as cells need to digest materials either of intracellular or extracellular origin (i.e., infectious agents escaping phagocytosis), but the autophagic machinery specifically engulfs cytoplasmic and not extracellular components. Instead, the engulfment of extracellular materials is a distinctive feature of the endocytic machinery, that begins with plasma membrane invagination and does not involve cytosolic cargoes. Another distinctive feature of autophagy is that the degradation occurs within lysosomes. Indeed, cells can also eliminate cytosolic proteins by degradation into proteasomes, but this process cannot be defined as “autophagic”, because it does not involve the lysosome as final degradative compartment [79]. Despite these common features, three autophagy mechanisms have been described: macroautophagy, chaperone-mediated autophagy (CMA) and microautophagy. They differ in terms of cargo selection and delivery mechanism into lysosomes. Further, current findings show that the autophagic machinery is also involved in the elimination of material outside the cell by secretion. Hence the definition of “secretory autophagy” has appeared in the scientific literature and currently it has become clear that secretory autophagy may not only represent a manner to discard waste material, but also a mechanism allowing the extracellular release of specific signalling molecules [80]. Although secretion is a destiny which is alternative to degradation within lysosomes and therefore “secretory autophagy” cannot be considered a proper autophagic response according to the criteria illustrated above, this mechanism appears of crucial importance for protein secretion, immune surveillance and cell signalling. Therefore, it is now clear that the decision whether to address material for degradation into lysosomes or to extracellular secretion is a key decision for the autophagic machinery, with pathological implications [81].

In this section, we are going to summarize the main features of different autophagy types, to describe the organelles involved in these processes and to discuss their possible role in secretory autophagy, including the crosstalk with exosome release. Moreover, we are going to review current knowledge on secretory autophagy in terms of cargo selection, molecular mechanisms and biological function.

### 4.2. The Autophagic Processes

The three autophagic processes characterized so far, although to a different extent, are macroautophagy, CMA and microautophagy. Macroautophagy has been so far the most investigated and characterized mechanism of autophagy. It is induced by several stimuli, such as nutrient deprivation (e.g., glucose, aminoacids) and other cellular stressors [82]. Macroautophagy begins with the cytosolic nucleation of a double membrane structure, whose possible origin has been extensively reviewed elsewhere [83]. The nucleating membrane continues to extend, leading to an open structure, the phagophore, which progressively surround cytoplasmic material. The process comes to an end with the closure of the membrane and the formation of the so-called autophagosome. In turn, the autophagosome fuses with lysosome to form an autolysosome, a degradative structure which allows the breakdown and recycling of macromolecular constituents. Lysosomes are than reformed from autolysosomes. The molecular machinery underlying macroautophagy has been extensively investigated [84] and a few points are useful to fix. From a molecular point of view, the autophagy process relies on a group of genes, originally identified as autophagy-related (Atg) by genetic screens in yeast. Most of them have homologs in mammals [85]. Briefly, in mammalian cells, upon autophagy induction, the Ulk1/Atg1 complex initiates the nucleation of the phagophore structure, recruiting the Beclin-1 complex, which carries phosphatidylinositol 3-kinase (PI3K) activity in its Vps34 component and promotes phagophore expansion via phosphatidylinositol-3, 4, 5-triphosphate (PIP3) synthesis. Subsequently, other Atg genes enters the scene. Two complexes with ubiquitin-conjugation properties (Atg5-Atg12 and Atg7-Atg3) are necessary to conjugate Atg8 protein (LC3 in mammals) to phosphatidylethanolamine (PE) on the growing membrane of autophagosome. Once autophagosomes are formed, they can either fuse with lysosomes, to form autolysosomes, or with organelles of endosomal origin, such as late endosomes, to form amphisomes [86]. Interestingly, a process called ALR (autophagic lysosome reformation) is necessary to allow the regeneration of lysosomes from autolysosomes [87,88].

CMA and microautophagy have been less characterized as compared to macroautophagy. Both processes are characterized by the relevance of HSPA8 (also known as Hsc70) molecular chaperone, which binds to cytosolic proteins carrying the KFERQ pentapeptide sequence for the selection of cargoes to be degraded into lysosomes. However, CMA and microautophagy differ for mode of cargo delivery into lysosomes: in CMA, the HSPA8/cytosolic protein complex is targeted to the lysosomal surface, where a specific isoform of the LAMP2 protein, LAMP2A (but not LAMP2B and LAMP2C) acts as a receptor. LAMP2A forms a multimeric cluster which translocates the cargo across the lysosomal membrane. In the lysosomal lumen, an isoform of HSPA8 binds to the cargo, retaining it within the lysosome and allowing its degradation by hydrolases [89]. Consequently, CMA does not involve the formation of vesicular structures. On the other hand, in microautophagy HSPA8 targeted entities are taken up by direct membrane invagination of either lysosomes (microautophagy) or endosomes (“endosomal microautophagy”) [90]. Therefore, in microautophagy cytosolic proteins are delivered to lysosomes within vesicles. This is distinctive feature as compared to the translocation complex of CMA, which additionally implicates the denaturation of the protein across the translocation complex [89]. It is interesting to underline that endosomal microautophagy closely resembles ILVs/exosomes biogenesis (see Section 3). Indeed, it has been demonstrated that endosomal microautophagy is ESCRT-dependent, whereas microautophagy is ESCRT-independent [91].

### 4.3. Non Degradative Function of Autophagy: Secretory Autophagy

Cells have a strategic alternative to lysosomal degradation in order to dispose garbage, i.e., its extracellular release. When this occurs in an autophagic machinery-dependent manner, the process is defined as “secretory autophagy”, although cellular entities are not degraded into lysosomes and therefore the mechanism could be not properly defined as autophagic [78]. Secretory autophagy has been initially identified during studies aimed at investigating how proteins lacking signal peptides and thus unable to enter Endoplasmic Reticulum (ER) can be released extracellularly, an occurrence known as Unconventional Protein Secretion (UPS) [92]. In eukaryotes, secreted proteins usually transit the ER and Golgi, then are released extracellularly upon vesicle fusion with plasma membrane, but protein lacking the signal peptide cannot exploit this route.

One of the earliest examples of protein released extracellularly by secretory autophagy has been interleukin 1β (IL1β). This is a proinflammatory cytokine lacking the signal peptide and localized in the cytoplasm as an inactive precursor. Upon inflammasome activation, IL1β is proteolytically processed by caspase-1 into its mature form, which is exported outside the cell, where it binds to its cognate receptor, evoking a pro-inflammatory response [93]. The export process of IL1β has been intensively investigated: initially, it was found that it took place through membranous carriers of unknown origin [94], but 20 years later it was discovered that the extracellular delivery of IL1β relies on an autophagy-based UPS mechanism [95] and stimulation of autophagy led to inflammasome-dependent IL1β secretion [95]. Nowadays, an additional export mechanism dependent on membrane pore formation has been also characterized [96].

In addition to IL1β, several proteins relying on autophagic machinery for secretion have been identified [97]. A leaderless protein whose release is prompted by inflammatory stimuli is the High-mobility group protein B1 (HMGB1) nuclear protein [98], that acts as a Damage Associated Molecular Pattern (DAMP) and whose autophagy-dependent secretion by keratinocytes plays a pivotal role in psoriatic skin inflammation [99]. Another relevant protein released by secretory autophagy is TGF-β1, which was demonstrated to be secreted through an unconventional pathway dependent on the autophagic machinery and cytoskeletal regulators [100]. Interestingly, membrane proteins have also been shown to be recruited to the plasma membrane by secretory autophagy. Indeed, specific autophagy and ESCRT components participate in cystic fibrosis transmembrane conductance regulator (CFTR) unconventional secretion, thus suggesting that the autophagic machinery may play a specific role in cystic fibrosis pathogenesis [101].

From a functional point of view, secretory autophagy is relevant for immune-related function and the autophagic process is important to get rid of intracellular pathogens, including bacteria and viruses. In normal conditions, pathogens enter cells via phagocytosis and are degraded upon phagosome fusion with lysosome to form a phagolysosome [102]. However, the degradation pathway may fail, and pathogens can escape the phagosome and enter the cytoplasm. In this condition, the autophagic machinery is activated and the pathogen became “the facto” a cytoplasmic material that is engulfed by the autophagosome double membrane. When this structure fuses with lysosomes for degradation, an autophagolysosome is formed [103]. For this reason, the terms autolysosome and autophagolysosome are not equivalent and should not be used indifferently [86].

The formation of an autophagolysosome may also occur through a different process. Indeed, phagocytosis may be accompanied by the formation of a single membrane phagosome that contains extracellular material or dead cells destined for degradation and recruits the key macroautophagy marker LC3B, a process known as LAP (LC3-associated phagocytosis) [104]. A relevant LAP feature is that LC3B is recruited not on a double membrane organelle like an autophagosome, but on a single membrane phagosome formed upon plasma membrane invagination. Moreover, LC3-labelled phagosome contains extracellular and not cytosolic material, as for the other forms of autophagy. LAP requires several components of the macroautophagic machinery, such as the Atg5-Atg12-Atg16L1 complex [105], the Atg3-Atg7 complex and the PI3K activity [106]. Conversely, other components of the macroautophagic machinery appear to be dispensable, such as Ulk1 [104], whereas some, such as the activation of the protein Rubicon, commit the cell to LAP, inhibiting autophagy [107]. LAP is involved in the response to pathogens [101], but also in the elimination of dead cells [108], including the finalization of the entotic process, by which cells engulf and cannibalize other cells [109].

Independently from how they are formed, autophagolysosome content is destined to degradation. However, this step may fail and the content of the autophagolysosome may be released extracellularly. In this manner, the cell may get rid of potential pathogens, but in some cases these pathogens can still be able to infect neighboring cells, thus spreading the infection [81]. It has been demonstrated that intracellular pathogens like Mycobacteria and Brucella spp. may be released extracellularly in this autophagy-dependent manner [110,111]. This occurrence indicates that the autophagic machinery may be important to avoid plasma membrane disruption during microbial egress. Interestingly, the impairment of lysosomal function causes the failure of autophagolysosomal content degradation. As for example, this impairment happens upon activation of the P2X7 purinergic receptor, leading to the extracellular release of autophagolysosomes by microglial cells [112]. Like bacteria, several viruses have shown the ability to exploit the autophagic machinery to exit infected cells. Enterovirus may exit cells exploiting phosphatidylserine-positive vesicle originating from autophagosomes [113,114] and their release does not necessarily implicate cell lysis. Similar findings were reported for poliovirus [115] and zikavirus [116]. Moreover, it has been widely reported that enveloped viruses hijack the endosomal-lysosomal pathway for biogenesis and infection [117]. As a matter of fact, EVs and viruses share common features in their size, biogenesis and uptake and, when released by infected cells, EVs have been demonstrated to contain viral components, such as proteins and genetic material [118].

The role of secretory autophagy is not only to allow pathogens to exit from cells without cell lysis, but also to help immune response by participating to the unconventional secretion of antimicrobial molecules. In the human body, epithelial cells provide not only a physical but also a chemical barrier to infection. In the gastrointestinal tract, the pathogen Salmonella typhimurium enters cell to initiate infection and induces autophagy in intestinal Paneth cells [119]. The characterization of the entry process has led to the discovery that the infection is associated with the formation of LC3-positive granules containing lysozyme surrounded by double membrane autophagic structures, thus indicating that upon infection the autophagic machinery engulfs lysozyme granules and release them in bulk through secretory autophagy. Consequently, when autophagy is inhibited, this type of antibacterial defense is impaired [120]. Interestingly, Atg16L1, an important autophagy gene, is a risk allele for Crohn’s disease, an Inflammatory Bowel Disease of the small intestine [121]. The loss of either Atg16L1 or Atg5 leads to severe reduction in granule exocytosis from Paneth cells upon Salmonella typhimurium infection and thus to a less efficient antibacterial defense. In turn, this contributes to the inflammatory condition that characterizes this pathology. In summary, autophagy is relevant for the intestinal epithelial function, not only because it degrades invasive bacteria, but also because its secretory function is fundamental for the response to microbial invasion at tissue level [122].

A large amount of work on autophagy secretory functions came from studies on proteins involved in the pathogenesis of Parkinson’s and Alzheimer’s disease, namely α-synuclein and β-amyloid precursor protein (βAPP). As for α-synuclein, it has been demonstrated that this protein, when the autophagic-lysosomal degradative pathway is impaired, is released extracellularly. Specifically, when the autophagic-lysosomal system is inhibited by bafilomycin A1 treatment, reduced intracellular α-synuclein aggregation and increased α-synuclein extracellular secretion is observed, either via exosomes [61,123] or microvesicles shedding [124]. More recently, it has been reported that autophagy inhibition promotes the release of α-synuclein via EVs with a hybrid autophagosomes-exosome-like phenotype and increases the number of amphisomal structures, thus suggesting that unconventional secretion of α-synuclein may occur not only via EVs, but also via secretory autophagy [125]. Similarly, the characterization of tubulin polymerization-promoting protein p25α has showed its co-localization with α-synuclein into autophagosomes. p25α lowers the mobility of autophagosomes and hinders their final maturation by preventing fusion with lysosomes, causing an increase in the basal level of α-synuclein secreted into the medium in an autophagy-dependent manner [126]. The elevated α-synuclein exocytosis promoted α-synuclein deposition and cell death in neighboring neurons [127]. This finding provides a potential link between autophagic dysfunction and the progressive spread of the pathology [117]. In the case of Alzheimer’s disease (AD), very early evidence for the role of MVBs in βAPP metabolism was provided by the study by Vingtdeux et al., which showed that βAPP and its catabolic derivatives are secreted in exosomes [62]. Later, further studies focused on exosomes as potential intercellular carriers of the pathogenic proteins βAPP and tau [128]. On the other hand, it has been demonstrated that exosomes are not the only manner to release the amyloid β (Aβ) peptide extracellularly, as Aβ is generated in autophagic vacuoles [129] and Aβ secretion/plaque formation have been shown to depend on autophagy [130]. In addition, the autophagy-mediated secretory pathway is responsible for both normal and pathological tau release in neurons [131]. The secretion from astrocytes of insulin-degrading enzyme, one of the major proteases of Aβ peptide, is also mediated by an autophagy-based unconventional secretory pathway [132]. Collectively, these findings indicate that exosomes and autophagosomes/amphisomes released upon secretory autophagy induced by lysosomal dysfunction may represent vehicles for the transfer of toxic proteins to other cells [67].

### 4.4. The Molecular Machinery underlying Secretory Autophagy

The molecular machinery underlying secretory autophagy is far from being satisfactorily elucidated, although a few studies have provided some evidence [97]. In 2010, a seminal study in yeast identified Acb1 as the first protein to be released by secretory autophagy [133,134]. This protein is an Acyl coenzyme A (CoA)–binding protein, homolog to ACBA in Dictiostelyum discoideum. The formation of CUPS (Compartments for Unconventional Protein Secretion) in yeast, which are similar to pre-autophagosomal structures called omegasome in mammals [62], can be considered the first step of “secretory autophagy”. Unconventional secretion of Acb1 depends on Atg proteins, which also control autophagosome formation, and for this reason the process has been initially named “secretory autophagy” [133,134]. Another yeast protein which is involved in this process is Grh1 [135], whose homolog in mammals is GRASP (Golgi Reassembly Stacking Protein). ESCRT proteins involved in MVBs formation and SNARE proteins fundamental for membrane fusion have also been implicated [133]. In the case of SNARE proteins, the SNARE Sso1 is necessary for plasma membrane fusion, whereas VAMP7/VAMP3, a SNARE complex involved in the fusion with vacuole, is not necessary for secretory autophagy, thus indicating that specific factors may regulate the secretion vs degradation pathway [133].

In mammalian cells, Dupont et al. [95] initially reported that the secretion of IL1β depends on Atg5, on the GRASP homolog GRASP65, and on the small GTPase Rab8A. GRASPs are involved in the regulation of cargo transfer to the Golgi, and so far, they are considered the best available markers for UPS [92]. In mammals, it was found that both GRASP55 and GRASP65 are necessary for secretory autophagy, but GRASP65 is a better marker, as GRASP55 is necessary also for degradative autophagy [136]. As for Rab8A, this regulator of polarized sorting to plasma membrane is necessary for secretory autophagy, whereas Rab8B is not, being involved in the maturation of the autophagosome for degradative purposes [95,126,137].

The mechanism of cargo selection for secretory autophagy is unclear. By analysing IL1β secretion upon lysosomal damage as a model system, Kimura et al. [138] found that several TRIM proteins are involved in IL1β secretion. TRIMs are a large family of proteins (about 80 members), known to function as cargo receptors during autophagy. Kimura et al. focused their attention on TRIM16, demonstrating that this protein is necessary for the secretion of IL1β, via the formation of a complex with galectin 8, a lectin that binds to sugars located on the luminal leaflet of lysosomal membrane and becomes exposed on the cytosolic leaflet upon lysosomal damage [138]. The TRIM16-dependent cargo is then addressed for secretion and not for degradation by the action of a combination of SNARE proteins, i.e., the R-SNARE Sec22 on autophagosomes, Syntaxin 3 and possibly 4 on plasma membrane, SNAP23 and SNAP29 [138].

Autophagosomes cannot only fuse with lysosomes to form autolysosomes or be released extracellularly via secretory autophagy, as they have a third option, i.e., to fuse with late endosomes or MVBs to form amphisomes. In turn, amphisomes are either addressed to degradation by fusion with lysosomes or released extracellularly [139,140]. These options are important because they indicate an important crosstalk between the autophagic and endocytic system. Due to their origin from autophagosomes, amphisomes contain typical autophagosome markers such as lipidated LC3, and due to their origin from endosomes, they contain endosomal markers such as Rab5, Rab7 and Rab11 [141], as well as small amount of V-ATPase [142]. Amphisome secretion may be functionally relevant. Upon induction of autophagy in epithelial lung cells by IFNγ treatment, annexin 2 is released by unconventional secretion, through a process dependent on Atg5, Rab11 and Rab27A. These findings indicate that the formation of autophagosomes, MVBs and their fusion with plasma membrane are all relevant processes for annexin 2 release and suggest the secretion of amphisomes carrying both exosome and autophagy markers. As in the case of IL1β secretion, this process requires Rab8A [143]. In 2012, a study by Griffiths et al. [144] provided evidence that mature reticulocytes internalize plasma membrane in glycophorin A containing vesicles. These structures fuse with autophagosomes before exocytosis and are released, thus indicating that amphisomes may be relevant for membrane remodelling events necessary for reticulocytes maturation. In goblet cells of the intestinal epithelium, amphisomes promote the secretion of mucins, that have a crucial role in providing the mucus barrier that protects against intestinal pathogens [145].

A few studies have tried to shed light on the relation between autophagy, secretory autophagy via autophagosome/amphisomes and exosome release. In particular, a few studies have shown that key proteins for the macroautophagy process such Atg5 are involved in non-autophagic function. One example is the stimulation of the vacuolar pumps dissociation from MVBs, that impairs MVBs acidification, prompts their fusion with the plasma membrane and leads to the release of exosomes [146]. In autophagy-deficient Atg5 knockout cells, the treatment with V-ATPase inhibitors in Atg5 knockout cells provided evidence that luminal pH plays a role in controlling whether MVBs must undergo fusion with lysosomes for degradation or with plasma membrane for exosomes release [147]. In another study, the Atg12-Atg3 complex was shown to interact with Alix, a protein involved in membrane fission which interacts with ESCRT components involved in exosome biogenesis. In the absence of the Atg12-Atg3 complex MVB morphology and trafficking were altered and exosome biogenesis reduced [148]. In summary, current investigations provide evidence that when different cell stressors inhibit autophagic degradation into lysosomes, autophagy-dependent secretion is activated in order to discard unnecessary/harmful material, whereas when autophagy works properly, the lysosomal degradation is the favourite option. Molecular mechanisms underlying these two possible destinies are not fully clear, and their elucidation is particularly relevant to shed light on age-related neurological disorders and lysosomal storage disorders, both characterized by lysosomal impairment [149].

## 5. Conclusions

Lysosomes have initially been considered as degradative compartments, but currently, there is evidence that they have secretory functions fundamental for cell homeostasis. Similarly, the autophagic pathways have been considered processes leading to the degradation of cellular waste upon fusion with lysosomes in order to recycle cellular components, but it is now emerging that intermediate compartments of the autophagic system such as autophagosomes may be exploited to release proteins lacking N-terminal peptide by unconventional secretion, as well as to get rid of infectious agents. Further, when exosomes originating from the endosomal system were initially described, they were considered a tool of dispose extracellularly unnecessary material, but later, it emerged that they carry functionally relevant molecules, and they are now considered an additional manner to transmit extracellular signal. The molecular machinery and the cross-talk underlying these events is only partially known. The elucidation of key factors responsible either for degradation or release of lysosomes, endosomes and related organelles/structures will be of great relevance, because garbage elimination is a fundamental target to alleviate pathologies characterized by intracellular accumulation of undigested substrates, such as lysosomal storage disorders and age-related neurological disorders.
